# Bone void filler materials for augmentation in comminuted fractures: a comprehensive review

**DOI:** 10.1186/s13018-025-05857-2

**Published:** 2025-05-08

**Authors:** S-Sina Mohammadi, Sunjeev S. Phull, B. Sonny Bal, Mark R. Towler

**Affiliations:** 1 Missouri University of Science and Technology, Department of Chemical and Biochemical Engineering, 1101 N State St, Rolla, MO 65409 US; 2 Orthopedic Surgery Aetna-CVS Health, Columbia, MO 65211 US

**Keywords:** Comminuted fracture, Augmentation, Bone Void filler, PMMA, Bone grafts

## Abstract

Comminuted fractures, characterized by multiple bone fragments, present significant challenges in orthopedic surgery. Effective treatment often requires augmentation techniques to enhance fixation stability and promote bone regeneration. This review explores the application of bone void filler materials, including autografts, allografts, polymethyl methacrylate (PMMA), and synthetic bone substitutes such as calcium phosphate ceramics and bioactive glass, in managing comminuted fractures. Autografts are the gold standard due to their superior osteogenic potential but are limited by donor site morbidity and availability. Allografts mitigate these issues but face concerns regarding immunogenicity and reduced biological activity. PMMA, widely used for structural augmentation, provides immediate stability but suffers from thermal necrosis, polymer shrinkage, and cytotoxic risks. Synthetic bone substitutes, including calcium phosphate cement and bioactive glass, offer promising alternatives by promoting bone integration while reducing complications associated with traditional grafts. However, their mechanical limitations and their artificial nature leave room for improvement. The review highlights recent advancements in biomaterial modifications to improve degradation rates, osteointegration, and mechanical resilience, such as composite materials and ion-doped bio ceramics. Despite these innovations, a gap remains in developing an ideal augmentation material that combines structural integrity with bioactivity. Future research should focus on integrating bioactive elements with load-bearing capabilities to optimize patient outcomes in comminuted fracture management.

## Background

Major car crashes, falling from a height, athletic injuries, or any other unprecedented impacts exerted on bones may lead to a fracture. In 2019, the global incidence of fractures was estimated to be approximately 178 million [1]; with 2.9 million patients suffering from femoral shaft fractures each year from traffic accidents [2]. From an engineering standpoint, a bone fracture occurs when trauma induces a failure in the bone's structural integrity, surpassing its load-bearing capacity.

**Fig. 1:**
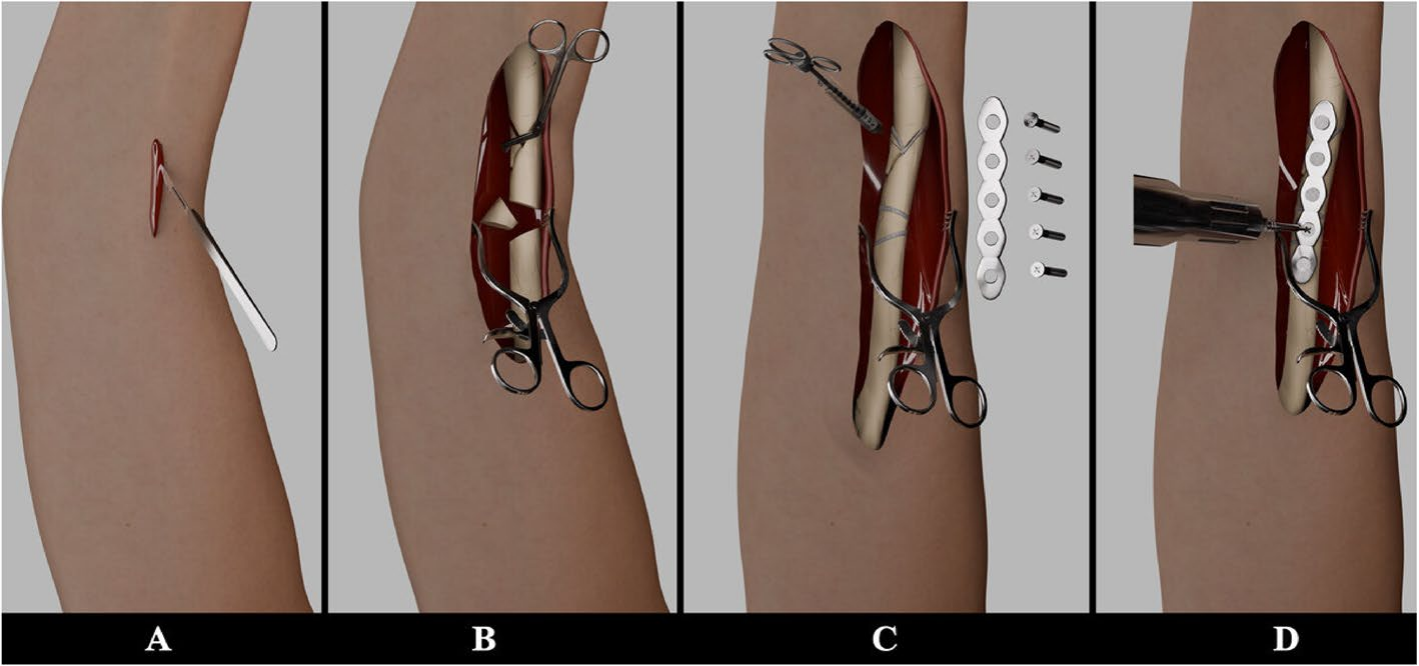
Illustration of ORIF surgery: **A**) Removing soft tissue covering the fracture site **B**) Aligning fractured bone in their anatomical axis **C**) Using bone cement to secure the bone **D**) Applying metallic implant for final fixation

Bone exhibits both an inherent regenerative capacity and a remarkable adaptability to its environment, actively contributing to defect healing through self-restoration and repair [3]. However, in some cases, relying on this ability is not always sufficient to fully recover from certain traumas. For instance, comminuted fractures are a type of trauma where the bone breaks into three or more segments and requires surgical intervention to assist with bone healing [4]. In such cases, comorbidities like soft tissue damage further complicate fracture repair [5]. Open reduction internal fixation (ORIF) is a surgical technique for comminuted fractures [6]. The procedure begins with open reduction, where the surgeon makes an incision at the fracture site to directly access and manually realign the broken bones to their normal position. Following this, the realigned bones are secured using internal devices such as metal plates, screws, rods, or pins allowing them to heal in the correct anatomical position (Fig. 1) [5, 7].

Depending on the severity and location of the fracture, orthopedic surgeons will choose between metallic and polymeric materials for prophylactic fixation. Metallic implants, such as titanium and stainless-steel alloys, are preferred for their superior mechanical properties [8], however, these materials can cause adverse reactions and corrosion, potentially leading to issues such as aseptic loosening and stress shielding, ultimately leading to revision surgeries. Aseptic loosening in cemented implants refers to the non-infectious failure at the bone-cement interface, often caused by stress and wear [9], while stress shielding can weaken surrounding bone due to uneven load distribution and mismatch of Young’s modulus between bone and implant [10]. In contrast, polymeric implants offer superior biocompatibility and biodegradability but are limited by their inferior mechanical properties [11].

**Fig. 2:**
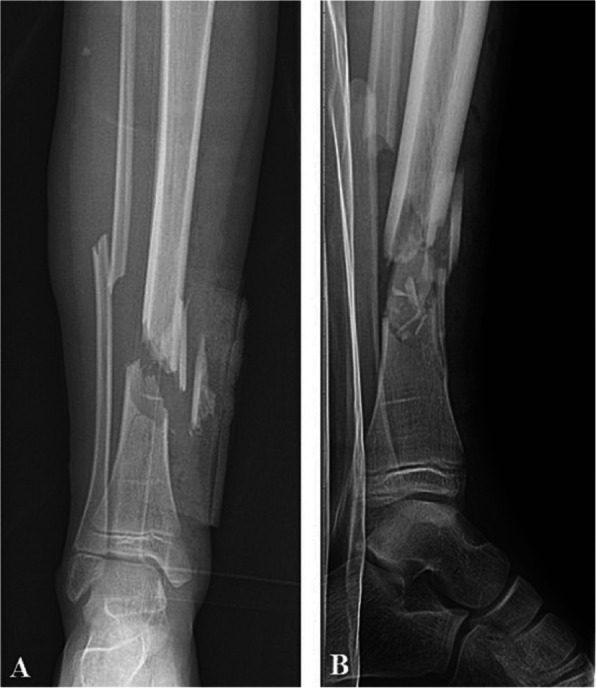
Example of comminuted fracture pattern of tibia fracture X-ray images in (**a**) anterior–posterior and (**b**) lateral view [13]

The complexity of comminuted fractures (Fig. 2) often results from high-impact trauma or even low-impact forces exerted on patients with chronic diseases (such as osteoporosis or metastatic bone disease). The fact that the bone is shattered into multiple segments complicates the process of achieving and maintaining proper alignment. As such, augmentation techniques, such as bone grafting or advanced biomaterials, are often necessary to enhance the fracture site's stability, facilitate proper healing, and restore the bone's anatomical structure. These materials support fixation devices and promote biological healing by filling defects and providing a scaffold for bone regeneration. A study by Yee et al*.* [12] examined the effectiveness of augmentation in 76 elderly patients who received an intramedullary nail device for femur comminuted fracture. Among these, 47 patients had augmented implants with polymethylmethacrylate (PMMA), while 29 had non-cemented implants, with a minimum follow-up period of 6 months. The results showed a fixation failure rate of 2.2% in the cemented implant group, which was significantly lower than the 13.8% failure rate observed in the non-cemented implant group and demonstrates the superiority of augmented reconstruction for comminuted fractures.

This review critically examines various bone void filler materials for augmenting comminuted fractures, focusing on bone grafts (autografts and allografts), PMMA, and synthetic bone substitutes like calcium phosphates and bioactive glass. Their mechanical properties, biological effectiveness, and clinical applications will be evaluated to determine their roles in enhancing the repair of comminuted fractures by promoting bone regeneration and addressing their limitations. Selected mechanical and rheological properties of commercially available products are summarized in Table 1.

**Table 1:** Commercially available augmentation materials outlining their mechanical and rheological properties and degradability

Commercial product (Augmentation type)	Degradability	Working Time (Mins)	Setting time (Mins)	Bending strength (MPa)	Compressive strength (MPa)
Cancellous iliac crest (Autograft) [14]	Yes	NA	NA	3.26	4.29
DBM (Allograft) [15, 16]	Yes	NA	NA	16.3 ± 6.8	2.9
Simplex® P (PMMA) [17, 18]	No	7	14.3	150	120
Euro Bone (CPCs) [18]	Yes	3–15	720	0.47	17
Calcemex® (PMMA + β-TCP) [19–21]	Partial (due to β-TCP)	5.45	NA	30	30.69 ± 1.97

## Bone grafts

Each year, over 2.2 million surgical procedures worldwide rely on bone grafts to enhance the repair of skeletal defects [22]. In certain fractures such as comminuted fracture patterns, the body’s natural healing process may be delayed or fall short, lacking sufficient functional cells (osteoblasts, osteoclasts, osteocytes), stability, vascularization, growth factors, and a growth matrix. In such cases, bone grafts provide the necessary support to enhance healing [23]. The ideal graft should exhibit four important characteristics: osteointegration, osteoinduction, osteoconduction, and osteogenesis. Based on their source, bone grafts are classified into three main groups: autografts, allografts, and xenografts. Although xenografts are used as orthopedic augmentations in Europe, for example, Tutobone® (Tutogen Medical GmbH, Germany) for hip socket and spinal applications [24], they are not approved by the FDA for skeletal applications [25]. Choosing the correct bone graft necessitates understanding the patient's specific bone healing needs, the properties of available graft options, and considering important factors such as the patient's age, mechanical stability, harvest-site morbidity, clinical outcomes, and cost [26].

### Autograft

Autografts are the gold standard bone grafts, which involve transposing a patient’s bone from a separate part of the body (such as the iliac crest or fibula) to the bony defect site. Such grafts possess the four characteristics of an ideal implant [27, 28]. Autografts are abundant in bone-forming and osteoinductive cells, providing a scaffold at fracture sites that facilitate the penetration and activity of blood vessels and osteoblasts [27]. However, autografts have limitations, including increased operative time, limited availability, significant morbidity from blood loss, wound complications, local sensory loss, and chronic pain. Up to 15% of patients experience donor site pain lasting over three months, with pain severity proportional to the extent of graft extraction [23, 29].

Autografts are classified, based on microstructure, into five categories: cancellous, cortical, vascularized, bone marrow aspirate, and platelet-rich plasma; of which cancellous and cortical are the most commonly used. Cancellous bone grafts are rich in osteoblasts and osteocytes and are widely used because of their superior osteogenic potential [30]. Their large trabecular surface aids revascularization. The grafting process involves resorption, substitution, and neovascularization, leading to new bone formation over 6 to 12 months [30].

Cortical bone grafts offer greater structural support compared to cancellous grafts but have limited osteogenic potential due to low cellular density and poor revascularization; moreover, their incorporation is primarily mediated by osteoclasts through creeping substitution, which is slow and can take years to completely integrate with the surrounding bone [30].

Biermann et al*.* [31] systematically reviewed the biomechanical assessment of augmentation in proximal humeral comminuted fractures using ORIF with plate fixation. They showed that autografts are the most efficient way of providing primary (soft callus formation) and secondary (hard callus formation) stability. The study found that using an autogenous fibular strut graft enhanced varus stiffness by 3.8 times and failure load by 1.7 times compared to constructs without augmentation, providing more rigid fixation under bending stress. Although the paper used a good qualitative approach for systematically reviewing the literature, it lacks sufficient quantitative data on clinical outcomes, limiting comprehensive comparisons and precise assessment of each augmentation method's effectiveness.

While autografting is the gold standard, its drawbacks, such as increased operative time and donor site morbidity, drive clinical researchers to seek more versatile and abundant alternatives.

### Allografts

Allografts are sourced from human cadavers or living donors and are used as augmentation and structural supports [22, 32]. Compared to autografts, allografts offer no donor site morbidity, multiple graft availability, and shorter operation times. However, they carry a minor yet notable risk of disease transmission. Zamborsky et al*.* [33] identified infection sources in bone allografts, including viruses like HIV (risk of 1 in 1.67 million), and Hepatitis type B and C viruses. Bacterial sources include Staphylococcus aureus, Clostridium, and Mycobacterium tuberculosis, with large grafts (massive allografts) having a bacterial infection rate of 11.7%, while smaller grafts (non-massive) carry a 0.7% risk. Although rare, prions and fungi also add risks, emphasizing the need for strict screening and sterilization. However, preservation and sterilization (techniques like freeze-drying) negatively impact the bone-forming potential of allografts by destroying osteogenic cells and deleteriously affecting their mechanical integrity [34, 35]. In a study by Dreyer et al*.* [36], the efficacy of autografts and allografts in repairing tibial defects was compared in normal and osteoporotic rat models. To determine the efficacy of grafts, defects were created in 24 female Norwegian brown rats’ tibiae, with autograft material harvested from bilateral tibiae during the defect drilling process and supplemented with tail vertebrae material in osteoporotic rats, then implanted and evaluated over 21 days. Rats were euthanized at the end of the study, and their tibiae were harvested and analyzed using micro-CT scans to assess bone volume fraction (BV/TV)—the ratio of newly formed bone volume to the total defect volume. The results showed that BV/TV for autografts was nearly 7% higher than allografts in normal bone and 16% higher in osteoporotic bone, reinforcing the superior bone-forming capabilities of autografts, particularly in compromised bone conditions like osteoporosis.

Based on their application, allografts can be categorized into three main types: cancellous, cortical, and demineralized bone matrix (DBM) [32]; the latter derived from allografts through sterilization, decellularization, and demineralization processes, which preserve growth factors, collagen, and non-collagenous proteins. DBMs exhibit enhanced osteoinductive potential compared to cancellous and cortical allografts due to the exposure of soluble factors post-demineralization [26, 37]. The demineralization process typically involves treating the bone with acidic solutions that dissolve the mineral content, particularly hydroxyapatite, which then uncovers the organic matrix rich in naturally occurring growth factors [38]. Soluble factors in DBMs enhance osteoinduction by exposing them to bioactive molecules that are critical for bone repair. Fibroblast growth factors aid angiogenesis and tissue repair, and transforming growth factor-beta enhances cell proliferation and matrix production. Insulin-like growth factors regulate cell growth and osteoblast differentiation, collectively boosting DBMs'effectiveness for bone regeneration compared to conventional allografts [39, 40]. The production of DBMs is costly due to required sterilization, precise decellularization and demineralization, strict quality control, and specialized storage, all ensuring bioactivity, safety, and regulatory compliance.

However, DBMs show sub-optimal mechanical properties [37]. For instance, Brink [41] compared the mechanical and biological properties of DBMs with allografts in trauma surgeries and showed that while both demonstrated osteoconductive properties, DBMs exhibited superior osteoinductivity, enhanced safety profiles, and higher levels of patient acceptance, though lacking concrete comparative data and relying on theoretical benefits, while allografts provided greater mechanical support, were more cost-effective, and had more extensive investigation in studies.

Kim et al*.* [42] conducted a study on augmented procedures and their impact on the surgical failure of proximal humeral comminuted fractures broken into 3 or 4 parts in 204 patients, all aged 65 years or older, by comparison of inferomedial screws with fibular allograft. They concluded that patients treated with fibular allografts demonstrated superior medial support, stability, and biomechanical properties such as load bearing and stiffness. The study also showed that fractures treated with grafts healed faster, with 3-part fractures uniting in 156 days (6 days sooner) and 4-part fractures in 159 days (9 days sooner) compared to non-graft treatments. While the paper provides valuable insights into the comparative effectiveness of fibular allografts, it falls short in offering robust quantitative data to substantiate its claims regarding biomechanical performance. Instead, it relies heavily on qualitative descriptions, which, though informative, lack the detailed numerical analysis needed to fully illustrate and validate the mechanical performance of the graft.

Allografts, especially in the form of DBMs, provide essential biological support in bone repair and significantly eliminate disease transmission, but are limited by strength and production costs. Synthetic options, including polymers and ceramics, offer greater availability and customizable properties to address specific clinical needs. Among synthetic materials, PMMA stands out as a widely used bone cement, valued for its mechanical strength, versatility, and ability to provide enhanced stability and customization in bone defect treatment, though it is not without its limitations.

## PMMA bone cement

From phone screen protectors to decorative items, PMMA was initially designed as Perspex® for airplane windows in World War II. Modifications in the 1940 s made it suitable for the dental industry, and since 1958, PMMA has become the gold standard bone cement in medical applications [43].

PMMA (or *acrylic cement*) is a versatile material in ophthalmology, dentistry, and orthopedics. In orthopedics, PMMA is utilized for hip arthroplasty, spinal fracture fixation, internal fracture-fixation plates (as a luting agent), and as a permanent bone substitute for treating pathologic fractures [8]. PMMA bone cement is widely used in orthopedic surgery due to its injectability, short curing time, low cost, early pain relief, and mechanical properties more similar to bone, suitable for the purpose of augmentation, such as Young’s modulus similar to bone (2.4–3.3 GPa) [44]. Moreover, PMMA’s viscosity allows it to fill irregular shapes and penetrate trabecular bone in its liquid form, allowing it to generate mechanical interlocking while hardening [43, 45]. On the other hand, PMMA is bioinert and does not bond with bone, leading to micromotion at the implant site, potentially causing osteolysis, aseptic loosening, or implant displacement [45]. Additionally, methyl methacrylate (MMA) monomers in PMMA bone cement may remain unpolymerized, potentially leaking into tissues and causing toxic effects like inflammation, allergic reactions, and cytotoxicity [44]. PMMA also exhibits thermal necrosis, as the exothermic polymerization reaches temperatures between 70 and 120 °C, which can cause collagen to denature and bone cells to die [46]. Furthermore, PMMA experiences up to 7% volumetric shrinkage during polymerization resulting in residual stresses of 4 to 24 MPa [47]. These residual stresses can create microcracks and compromise the stability of the surrounding bone. This stress may lead to implant loosening, hinder the bone's natural healing, and cause additional pain or complications, ultimately increasing the likelihood of graft or implant failure [48].

Ramanathan et al*.* [44] reviewed various modifications, fabrication methods, and additives aimed at improving the properties of PMMA, emphasizing the necessity for further long-term biocompatibility testing and optimizations. Their paper highlighted several strategies to enhance the properties of PMMA and overcome its drawbacks in orthopedic applications. To reduce the high exothermic temperature during polymerization, PMMA was combined with microcapsules containing phase-change materials up to 20wt% of total cement, such as paraffin, which effectively absorbed heat and lowered the maximum exothermic temperature, thereby minimizing thermal necrosis. To improve the bioactivity and osseointegration of PMMA, researchers incorporated bioactive components like hydroxyapatite, carbon nanotubes (CNTs), and natural polymers like chitosan, which enhanced bone formation and integration with surrounding tissues. Wang et al. [49] demonstrated that incorporating Mg–Al-layered double hydroxide micro sheets into PMMA significantly enhanced thermal insulation and released magnesium ions, which stimulated osteogenesis and improved osseointegration, resulting in a 2.17- to 18.34-fold increase in bone growth in a rabbit model. Ramanathan [44] also mentioned that the addition of CNTs and monticellite reinforced the mechanical properties of PMMA, increasing its resistance to cracking and mechanical stress. Surface modification using lactoferrin and UV irradiation also improved cell adhesion, proliferation, and extracellular matrix mineralization, improve the bioactivity of PMMA. The authors suggest that a multifaceted approach could produce an improved version of PMMA; however, they do not address the challenges of manufacturing this multi-step modified PMMA or the overall effectiveness of these complex modifications. The study also failed to address PMMA's lack of biodegradability, making it unsuitable for temporary fixation or procedures. Additionally, the paper’s emphasis on using nanomaterials, particularly CNTs, raises concerns about their long-term biocompatibility due to potential cytotoxic and genotoxic effects [50, 51]. Furthermore, many of the proposed strategies aim to reduce the release of unreacted MMA monomers indirectly, rather than fully preventing it. This limitation underscores the need for safer and more effective alternatives to enhance the performance of PMMA while minimizing the associated carcinogenic risks.

Goodnough et al*.* [52] systematically reviewed geriatric intertrochanteric femur fractures using PMMA and calcium phosphate cement (CPC) augmentation in various orthopedic situations, including comminuted fractures, suboptimal reduction quality, and poor bone quality cases. They concluded that PMMA-augmented fixation in unstable fracture patterns (comminuted) lowered the rate of mechanical failure and decreased hip pain compared to non-augmented fixation. They also showed that both PMMA and CPCs increase stability and biomechanical properties. However, the study focused on qualitative assessments, lacking quantitative data that could provide a more precise comparison of their clinical effectiveness.

**Fig. 3:**
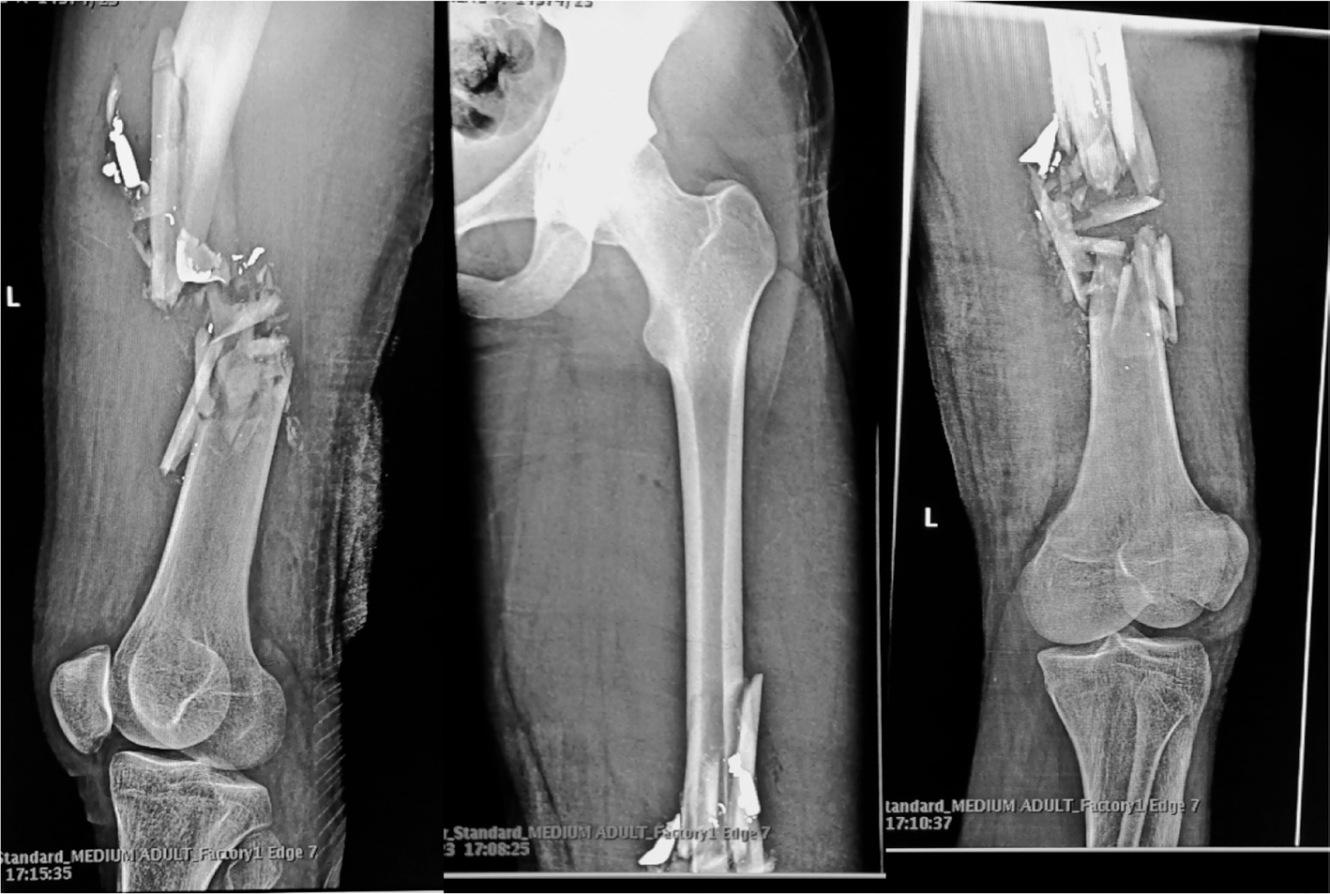
Lateral shoot-through X-rays of the left femur show severe comminution with sharp spicules involving the distal diaphyseal shaft, characteristic of high-energy trauma. Management of this type of unstable fracture typically involves intramedullary locked nailing, with augmentation using PMMA and autologous bone grafts to enhance mechanical stability and support bone regeneration [54]

In cases of comminuted fractures, the trauma can sometimes be so severe that surgeons have no choice other than using PMMA to reconstruct the bone and maintaining its integrity essential for subsequent implantation of metals or other fixators. Figure 3 illustrates a severely comminuted fracture, representing a typical case in which PMMA is required to aid in realignment and structural stabilization. Although PMMA has several disadvantages, it remains the gold standard bone cement due to the lack of other suitable materials with a long clinical history, near-bone mechanical properties, and affordability. In a study by Yang et al*.* [53] evaluated failure rate of PMMA in 31 patients (19 males and 12 females) who underwent two-stage revision hip arthroplasty with PMMA spacer for periprosthetic joint infections. The median age of the cohort was 56 years (interquartile range: 50–71 years). Among these, 45% experienced at least one spacer-related mechanical complication during the interim period. Specifically, 19.4% had spacer dislocations, 9.7% suffered spacer fractures, and 3.2% encountered both dislocation and fracture. Younger patients were significantly more likely to experience mechanical complications, with the study identifying age as a notable risk factor. Researchers continue to explore alternative bone substitutes to achieve improved augmented fixation. Among these, synthetic bone grafts have emerged as a promising solution, designed to replicate the natural properties of bone while addressing the limitations of traditional materials. These advanced substitutes, including ceramics and bioactive composites, demonstrate significant potential for enhanced integration and regeneration in bone defect treatment.

## Bone substitutes (synthetic bone grafts)

Biological grafts, particularly autografts, are often considered ideal for bone reconstruction due to their compatibility and effectiveness. However, their limited availability and associated complications restrict their use. A fourth category of grafts has been engineered to overcome these limitations by integrating the biological efficacy of natural grafts with the functional benefits of synthetic materials. Bone substitutes offer a longer shelf life, abundant availability, elimination of the risk of biological disease transmission, and the ability to be tailored for specific biological roles at the defect site [28]. Among all the materials, calcium phosphate ceramics, calcium phosphate cement, and bioactive glass are the ones used in comminuted fractures.

### Calcium phosphate ceramics

Calcium phosphate ceramics, mainly tricalcium phosphate, hydroxyapatite (HA), and biphasic calcium phosphate, are commonly used as they are similar to biological HA, the mineral phase of bone. Among these, β-tricalcium phosphate (β-TCP) is considered the gold standard [55]. These ceramics offer several advantages, including a porous structure that promotes vascularization, coordinated degradation with bone formation, radiopacity, and low immunogenicity during absorption. However, their inferior mechanical properties limit their use to non-load-bearing applications [56].

Scientists have suggested combining β-TCP with PMMA as a solution to enhance mechanical properties, with higher amounts of PMMA improving the overall performance of the bone substitute [57, 58]. Maluta et al*.* [20] evaluated the biocompatibility, osteointegration, and biomechanical properties of Calcemex® (TECRES, Italy) a cement composed of 48.6 wt.% PMMA and 50.1 wt.% β-TCP using saline solution to act as an emulsifier tested in a pig model. They directly drilled into the pig’s femur bone, implant the cement and maintained it for an experimental period of one year. The porcine model was sacrificed, and they cut the target bone, stained it, and demonstrated that Calcemex® had excellent integration with surrounding bone tissue, minimal inflammatory response, and maintained mechanical stability under weight-bearing conditions (compressive strength of 53.5 ± 5 MPa). The results suggest that Calcemex® has potential as a bone substitute with promising osteoconductive properties. However, the authors failed to assess the exothermic and volumetric shrinkage of these materials with no clear control sample as testing criteria for the effectiveness of the new Calcemex®. Moreover, the study lacks flexural strength assessment, a critical property for bone cement durability under bending and shear forces in vivo.

HA, as the mineral phase of bone, involves designing void fillers with the ability to mimic the bone's structure. Artificial and semi-artificial HA (contain natural HA combined with synthetic materials) are the prevalent choices that are frequently employed either as a coating or integrated within the implant structure like citric acid HA void filler [59]. As a synthetic bone graft material, HA provides osteoconductive properties and exhibits decent mechanical properties. These include a bending strength ranging from 38 to 250 MPa, compressive strength between 120 and 150 MPa, and a tensile strength of up to 300 MPa [60]. Despite its ability to mimic bone structure, synthetic HA has several limitations: In a study, Ielo et al. [61] reviewed HA-based bio-composites and highlighted their limitations in orthopedic applications, including low bonding with bone and inflammatory risks, which could potentially prolong recovery; however, they did not provide tangible data to support these claims. In addition, the material's brittleness renders it unsuitable for load-bearing applications and limits its vast clinical use [62].

Pokhrel [63] reviewed different HA composites, preparation methods, and their applications. The research showed that HA in the form of nanoparticles offers more active surface area allowing it to better integrate with bone, yet further research is needed to produce HA more efficiently. Alorku et al*.* [64] reviewed the challenges in HA production, emphasizing that traditional synthesis methods rely on costly, non-eco-friendly reagents such as ethylenediaminetetraacetic acid and polyvinylpyrrolidone. This approach often involves complex chemical processes that specific conditions such as maintaining a controlled pH, often around 9, using aqueous ammonia, and applying hydrothermal treatment at elevated temperatures (e.g., around 100 °C) to encourage the formation and stability of HA crystals​, to ensure HA purity and crystallinity, leading to environmental and economic inefficiencies.

Arifin et al*.* [65] evaluated the effectiveness of processed bovine hydroxyapatite (BHA) as an alternative to autograft and allograft in 56 trauma patients, ranging in age from 20 to 60 years, who had single bone fractures and complex non-union fractures (including 14 with a comminuted fracture). Radiological imaging showed that 80.36% of cases achieved union with full functional recovery, indicating that BHA effectively supports bone healing by filling bone defects. This outcome suggests that BHA is a promising alternative to traditional bone grafts, offering advantages such as greater availability and lower complication rates. However, the study also found that 19.64% of patients did not reach full functional recovery, highlighting the need for further research to confirm BHA’s long-term effectiveness. Additionally, a significant challenge with using HA from animal sources is the risk of immunological reactions, underscoring the importance of xenograft and immunology studies to validate its safety and efficacy.

CPCs retain the advantages of the ceramic form of calcium phosphate while also enabling clinicians to inject and mold it into irregular shapes before it hardens in situ [66]. Based on the final product of the setting reaction, CPCs can be categorized into two groups: brushite [CaHPO_4_ 2H_2_O] and apatite [Ca_5_(PO_4_) OH] [67]. CPCs have inferior mechanical properties, including tensile and compressive strengths, and are brittle, limiting their usage to non-load-bearing applications. This brittleness stems from their ceramic composition, which lacks the flexibility and toughness needed to absorb stress and resist fracture, making CPCs unsuitable for load-bearing sites where durability under repeated stress is essential [68]. The effect of weak mechanical properties can be seen in the aseptic loosening and micromotion of plates in comminuted fracture sites [69]. Additionally, the degradation characteristics of CPCs are inconsistent and debated, with their degradation rate often failing to align with the bone formation process. For instance, studies have shown that apatite-forming CPCs like Norian SRS® (DePuy Synthes, USA) exhibit minimal degradation even after 6 months, with only marginal signs of bone formation within cement cracks.

In contrast, brushite-forming CPCs like Biobon® (Graftys, France) display rapid degradation, achieving up to 96% resorption within six months, sometimes outpacing bone regeneration and resulting in fibrous tissue formation around the defect site​ [70]. However, CPCs'high biocompatibility and bioactivity continue to drive scientific advancements to improve their properties [71].

Liu et al*.* [72] reviewed various types and modifications of CPCs designed to adjust degradation rates and enhance mechanical properties. For example, to strengthen CPCs mechanically, poly(l-lactide-co-glycolide) (PLGA) acid nanofibers were incorporated into brushite cement, which not only promoted bone regeneration but also improved mechanical durability. Brushite CPCs typically exhibit compressive strengths between 10 to 40 MPa, whereas HA-based CPCs achieve strengths up to 60 MPa, making them comparable to cancellous bone. Moreover, inhibiting phase conversion to HA using magnesium ions or pyrophosphate prevented slow degradation and enhanced the resorption rate of brushite cement. Monetite cement, an anhydrous form of brushite, was also explored for its optimized degradation rate and balanced resorption, providing good mechanical stability while avoiding transformation into HA. Authors suggested that among all CPCs, modified anhydrous brushite with adjusted composition can align the need for bone cement in non-load bearing applications with degradation of 42% of cement when 43% of bone is formed, helping to maintain the required mechanical stability for new bone to grow. They focused solely on the degradation rate without considering the influence of mechanical forces on the implant, which is an uncommon scenario in the human body. The brittle nature of modified anhydrous brushite makes it prone to cracking, rendering it unsuitable for real-world conditions. In addition, the long setting time problem of CPCs is not evaluated in their experiment.

Demir-Oguz et al*.* [73] reviewed various methods to enhance mechanical and rheological properties such as reinforcing CPCs with PLGA, PLA, and bioactive glass, altering the microstructure through particle size and porosity changes, adjusting the liquid-to-powder ratio, and increasing bioactivity by adding growth factors and ions. Their results showed that the addition of 5wt% of bioactive glass (75wt% SiO₂ and 25wt% CaO) increased the compressive strength from 21.52 ± 2 MPa to 30.17 ± 1 MPa. They concluded that glass-incorporated CPC is a versatile bone cement that can be tailored to match the bone healing process and improve mechanical properties.

Egol et al*.* [74] studied the impact of CPCs on reducing screw penetration, where a screw unintentionally breaches the cortical bone or joint space, inside the bone and gradual bone fragment compression post-initial fixation (known as fracture settling). They studied 92 patients aged 22 to 84 years with 2-, 3-, and 4-part proximal humeral comminuted fractures treated with ORIF. The patients were divided into three groups: no augmentation (36 patients), allograft cancellous chips (29 patients), and CPC (27 patients). CPC significantly reduced fracture settling (compression of bone fragments during the healing process), with a mean settling of 0.07 mm at 3 months compared to 1.2 mm for no augmentation. Additionally, CPC completely prevented screw penetration whereas 19% of the no augmentation group and 14% of the cancellous chips group experienced this complication. The study provides valuable insights into CPCs for proximal humeral fracture augmentation. However, its retrospective design, small sample size, and lack of direct biomechanical analysis weaken its claims of mechanical superiority. Future research should include biomechanical testing to substantiate CPC's mechanical performance and clinical advantages.

Calcium phosphate ceramics like β-TCP and HA offer strong osteoconductive properties and good injectability in the form of cement but are limited in load-bearing applications due to weak mechanical properties. Advances such as β-TCP/PMMA composites and alternatives like BHA show promise, but further research is needed to optimize performance and address existing challenges.

### Bioactive glass

Glass is an amorphous, non-crystalline solid material typically composed of silica (SiO₂) along with various metallic oxides, formed by cooling molten components in a way that prevents the formation of a regular crystal structure [75]. Bioactive glass (BAG) is a calcium-rich material that initiates HA formation upon contact with body fluids and integrates seamlessly into the bone structure [76]. Before Bioglass®, integrating the implant with the direct bonding to the bone was a dream. Larry Hench engineered the composition of 45 wt.% SiO_2_, 24.5 wt.% Na_2_O, 24.5 wt.% CaO, and 6 wt.% P_2_O_5_ (45S5 glass) by melt quenching and designed the first-ever implant with the capability to integrate into the bone structure [77]. Later The sol–gel method enabled researchers to produce BAGs at relatively lower temperatures while offering precise control over the microstructure by adjusting pore size during the gelation and drying phases. This process also results in a larger available surface area [78]. BAGs are primarily utilized as synthetic bone grafts in orthopedics and periodontics [79]. They play a crucial role in bone regeneration and healing, particularly in addressing bone defects caused by trauma or the removal of tumors [79].

One of the most interesting fortes of BAG is the incorporation of different ions that can elicit a variety of biological effects (Table 2) As it integrates into bone, the BAG releases these ions into the surrounding environment and accelerates the healing process [80]. Silica-rich glasses (such as 45S5 glass) have a slow and incomplete degradation compared to HA-like materials, potentially leaving SiO_2_ in the scaffold which long-term complications are unclear. Additionally, its rigidity reduces malleability, which clinicians find impractical [78, 81].

**Table 2:** Potential ions that could be incorporated in different ceramic materials such as glass and ceramic-cement structure and their biological roles elicit in vivo

Name of ion	Biological Roles
Ca	React the with Body fluid and convert to HA [82]
Ga	Anti-cancerous affect/limit osteoclastic bone activity/antibacterials and anti-inflammatory [83]
Sr	Osteoporosis treatment with dual anti-resorptive and anabolic effect (promote osteoblast) [84]
Ag	favor the breakdown of microorganism resistance, antibacterial agent [85]
Cu	Angiogenesis, cell proliferation [86]
Zn	Anti-bacterial and Anti-inflammatory, enhance Osteoblast and inhibit osteoclast cells [83]
Fe	boosts energy production in cells and promotes the activity of genes involved in bone formation [87]
Mg	Important in human health in terms of brain, heart, and skeletal muscle health. Mg deficiency leads to more brittleness and, a decrease in bone mass density [88, 89]
Mn	Manganese regulates bone remodeling, supports matrix synthesis, reduces oxidative stress, and promotes bone formation in inflammatory conditions [87]
Si	17 to 20 ppm of soluble Si and 88 to 100 ppm of soluble Ca ions are required for the formation of mineralized bone tissue [90]
P	Increase the bioactivity by the formation of HA layer [91]
Ta	promoting osteoconduction and osteoinduction/Good mechanical properties [92]
B	Bone growth and maintenance, immune function, and psychomotor skills [84]

Granel et al. [93] explored several approaches to enhancing BAGs for biomedical applications. These include modifying the composition by changing the glass backbone from silicate to borate or phosphate to improve degradability, developing composite and hybrid materials to enhance mechanical properties, and doping BAGs with elements such as Sr, Mg, Cu, and Zn to elicit specific biological responses in vivo*.* These modifications aim to optimize BAGs for diverse medical uses, addressing both functional and biological requirements. They concluded that sol–gel-derived bioactive glass exhibits enhanced bioactivity, primarily due to its higher surface area and the potential for doping with various ions, which can address specific issues (for example, copper ions can enhance angiogenesis). However, these materials generally lack sufficient clinical validation and require a further clinical assessment to establish their effectiveness to establish their effectiveness fully.

In a study by Pernaa et al. [59], bioactive glass S53P4 (BAG-S53P4) and autograft bone were evaluated as substitutes for treating depressed lateral tibial plateau fractures. In a randomized, 11-year follow-up of 29 patients (14 with comminuted fractures) aged 38 to 65 (average age 52), outcomes were assessed for both groups. The findings showed no significant difference in functional or radiological outcomes. However, only 2 of 5 patients in the BAG group showed signs of progressive osteoarthritis, compared to 8 of 10 in the autograft group. BAG-S53P4 was well tolerated without adverse effects, supporting its effectiveness as an alternative to autografts for comminuted fractures, especially with subchondral bone defects. Despite this, there is no widely used commercial bioactive glass product for standalone augmentation; it is often used to increase the bioactivity of other implants.

## Conclusion

Comminuted fractures, particularly those with severe soft tissue damage, present complex challenges that often necessitate augmentation techniques to enhance stability and promote healing. Various materials, including autografts, allografts, PMMA bone cement, and synthetic bone substitutes, are employed to fill voids and support bone regeneration. Autografts, while considered the gold standard, are limited by donor site morbidity and availability. Allografts, despite their abundance, carry risks of disease transmission and reduced biological activity due to preservation methods. Synthetic bone substitutes, though mitigating issues related to availability and disease transmission, face limited clinical adoption due to suboptimal mechanical properties and insufficient data on their long-term performance. For example, CPCs lack the mechanical robustness required for high-stress environments, and BAGs, while promoting regeneration and ion integration, are not suitable for structural augmentation. PMMA, though providing immediate mechanical stability, is associated with cytotoxic risks, including thermal necrosis, polymer shrinkage, and MMA release. Despite these drawbacks, PMMA remains widely utilized due to its affordability, near-bone mechanical properties, and long clinical history.

Current research in this field often prioritizes qualitative analyses over quantitative assessments, particularly regarding mechanical properties such as flexural strength. This lack of robust mechanical characterization hinders the development of optimized materials tailored for the unique challenges of comminuted fractures. Moreover, there is a pressing need for studies that specifically address the complexities of comminuted fractures, focusing on tailored solutions that balance bioactivity with mechanical stability.

An ideal alternative for augmentation in comminuted fractures should combine the bioactivity and regenerative capabilities of autografts with the mechanical stability and affordability of materials like PMMA, while addressing the limitations of current substitutes. Future research should emphasize both the development of targeted solutions for comminuted fractures and the generation of comprehensive quantitative data to enable informed clinical decisions and material optimization.

## Data Availability

No datasets were generated or analysed during the current study.
